# Rehabilitation Strategies for Post-fasciotomy Recovery in Acute Spreading Cellulitis: A Case Report

**DOI:** 10.7759/cureus.68656

**Published:** 2024-09-04

**Authors:** Subrat N Samal, Bharat K Rathi, Nandini C Baheti

**Affiliations:** 1 Musculoskeletal Physiotherapy, Ravi Nair Physiotherapy College, Datta Meghe Institute of Higher Education and Research, Wardha, IND; 2 Sports Physiotherapy, Ravi Nair Physiotherapy College, Datta Meghe Institute of Higher Education and Research, Wardha, IND

**Keywords:** physiotherapy intervention, split screen grafting, rehabilitation, fasciotomy, cellulitis

## Abstract

Cellulitis is a skin condition that affects both the dermis and the subcutaneous fat. Acute compartment syndrome has been associated with streptococcal infection. The present case highlights the role of physiotherapy in rehabilitating a patient suffering from compartment syndrome due to cellulitis. A 45-year-old female complained of swelling over the left forearm for five days. After being referred to the surgery department, she underwent clinical examinations, indicating cellulitis. As part of the surgical procedure, she had a fasciotomy and split skin grafting. Her pain was assessed using the numerical pain rating scale (NPRS), gradual in onset and progressive in nature, and aggravated on movement. The patient's case demonstrates the importance of following a planned physical therapy treatment regimen to restore functional activity, range of motion (ROM), and muscle strength.

## Introduction

Acute spreading cellulitis is an infection of the skin, with the infection extending to the dermis and the subcutaneous fat. There are basically two types of cellulitis: purulent cellulitis and non-purulent cellulitis. Purulent cellulitis is manifested by pustules, abscesses, or purulent drainage. Management for purulent cutaneous infection mainly comprises drainage, with antibiotics playing an adjuvant role. It is, therefore, probably more correct to consider that purulent cellulitis is a surrounding inflammation of the site of infection [1]. Cellulitis is a bacterial infection of the skin, which at times is caused by pathogens after invading the skin through fractures. Streptococcus pyogenes, more popularly called group A Streptococcus, is a Gram-positive bacteria causing one of the most common diseases in humans and the pathogen responsible for many cases of cellulitis. Acute compartment syndrome has been associated with streptococcal infection, which is rare but likely. Such patients are treated with immediate surgical and intravenous antibiotic management [2]. Acute dermo-hypodermitis, or non-necrotizing cellulitis due to haemolytic streptococcus, is the cause of cellulitis (erysipelas). The major risk factor for the development of cellulitis is the presence of lymphoedema [3]. Lymphoedema was the major risk factor associated with cellulitis [4].

Soft tissue infections are one of the common pathologic diseases in clinical practice, ranging from skin to deep soft tissue and bone involvement. A timely diagnosis is essential to the care strategy so as to prevent complications and future progression. Even an excellent physical examination is insufficient to approximate the extent and character of a soft-tissue infection, although clinical diagnosis is the most common means by which to diagnose them. Under these circumstances, imaging becomes crucial in differentiating various patterns of soft tissue infection, displaying the extent of disease, and localizing collections that may be intervened with [5]. One common surgical technique is a fasciotomy. It relieves pressure or tension from the fascia, which is a layer of connective tissue covering the muscles. This could be of special importance if a patient suffers from compartment syndrome, the pressure inside a space containing muscles, typically exerted on the soft tissues, bones, and nerve structures, and causing severe tissue injury and necrosis in severe cases. Cellulitis is a serious bacterial infection affecting the skin and subcutaneous tissues, which can provoke considerable inflammatory responses and may result in elevated pressure within the muscle compartments of the upper limb [6,7]. In critical instances, surgical procedures such as fasciotomy may be necessary to alleviate this pressure and avert additional complications. Although skin grafting techniques, including mesh grafting, are important for wound coverage in low- to middle-income countries like India, they may not be the primary consideration in the management following fasciotomy. The scarcity of research on mesh grafting in resource-limited environments underscores the necessity for further investigation into diverse surgical and post-surgical management approaches [8].

After a fasciotomy, various complications may arise in the affected region, including oedema, infection, diminished blood flow, and the formation of haematomas. The occurrence of haematomas is especially prevalent as a result of bleeding that can take place within the tissues during the surgical procedure. Any physical stress, whether it is a squeeze or straining of grafted tissue, might be harmful. In order to avoid this type of complications, the transplant should be managed for a minimum period of 15 to 30 days. The integration of physical therapy and ultrasound massage can provide significant advantages for individuals experiencing deformities, helping to preserve the integrity of their skin for an extended duration. This has been aptly elucidated [9]. Following surgery for split skin grafting, patients reported a decline in all overall functional activities, which may comprise reduced mobility and strength as well as restriction in functional activity.

The aim of the study was to make the patient functionally independent by improving the range of motion (ROM), mobility, and strength of the musculature surrounding the joint and to make the patient perform activities of daily living effectively.

## Case presentation

Patient information

A 45-year-old woman presented with a complaint of swelling in her left forearm that had persisted for the last five days. She had been in her normal state of health until 15 days prior, when she experienced a cut injury to her left forearm, which resulted in pain and swelling. After consulting a local physician, she received medication that alleviated the pain; however, the swelling continued to worsen. Consequently, she was referred to the surgical department, where a clinical examination and various diagnostic tests, including ultrasound and blood tests (such as complete blood count and C-reactive protein), were performed. The results indicated cellulitis localized to the left forearm, particularly affecting the skin and subcutaneous tissues in the anterior compartment. Given the elevated pressure and potential for compartment syndrome, a split-thickness skin grafting procedure accompanied by fasciotomy was carried out. Following this, the patient was referred to the physiotherapy department to enhance the range of motion, muscle strength, and functional capabilities of the wrist, forearm, proximal interphalangeal (PIP), and distal interphalangeal (DIP) joints.

Clinical findings

The patient's verbal agreement was obtained, and a full assessment was performed. The patient was ectomorphic in built. The pain was examined using the numerical pain rating scale (NPRS), which was 07/10 at rest and 09/10 during exercise, with a gradual onset, progressive nature, and increased by movement. The pain was dull aching in nature, and it was eased by taking drugs. Grade 2 tenderness was observed at the suture site. The wrist, forearm, metacarpophalangeal (MCP), and interphalangeal (IP) joints all had a limited range of motion. ROM and manual muscle testing assessment for all the joints are shown in Tables 1-2, respectively.

**Table 1:** Pre-rehabilitation range of motion. MCP: metacarpophalangeal.

Range of motion	Right	Left
Wrist flexion	0–60^◦^	0–35^◦^
Wrist extension	0–60^◦^	0–30^◦^
Ulnar deviation	0–30^◦^	0–15^◦^
Radial deviation	0–20^◦^	0–10^◦^
1^st ^MCP flexion	0–50^◦^	0–30^◦^
2^nd^ MCP flexion	0–80^◦^	0–45^◦^
3^rd^ MCP flexion	0–75^◦^	0–40^◦^
4^th^ MCP flexion	0–80^◦^	0–40^◦^
5^th^ MCP flexion	0–85^◦^	0–45^◦^
1^st^ MCP extension	50–0^◦^	30–5^◦^
2^nd^ MCP extension	80–0^◦^	45–5^◦^
3^rd^ MCP extension	75–0^◦^	40–5^◦^
4^th^ MCP extension	80–0^◦^	40-5^◦^
5^th ^MCP extension	85–0^◦^	45–5^◦^
Forearm supination	0–80^◦^	0–70^◦^
Forearm pronation	0–80^◦^	0–70^◦^

**Table 2:** Pre-rehabilitation manual muscle testing. MCP: metacarpophalangeal.

Muscles	Right	Left
Wrist flexors	5\5	2-\5
Wrist extensors	5\5	2-\5
Ulnar deviators	5\5	2-\5
Radial deviators	5\5	2-\5
1^st^ MCP	5\5	3\5
2^nd^ MCP	5\5	3\5
3^rd^ MCP	5\5	3\5
4^th ^MCP	5\5	3\5
5^th^ MCP	5\5	3\5
Forearm pronators	5\5	2\5
Forearm supinators	5\5	2\5

Clinical investigations

In order to assess the severity and details of the cellulitis more thoroughly, further clinical investigations were conducted. A culture was obtained from a drain placed in the left forearm, which indicated the presence of methicillin-sensitive coagulase-negative staphylococcus. Additionally, a triplex colour Doppler examination of the left upper limb demonstrated evidence of cellulitis accompanied by subcutaneous oedema in the left arm and forearm while also confirming that both venous and arterial flow remained normal.

Physiotherapy intervention

As illustrated in Table 3, the physiotherapy protocol was made to restore range of motion and strength and improve the quality of life.

**Table 3:** Physiotherapeutic management. NA: not applicable; ROM: range of motion; MCP: metacarpophalangeal; DIP: distal interphalangeal; PIP: proximal interphalangeal.

Problem list	Goals	Intervention	Dosage (5 times a week for 4 weeks) [10]	Rationale
N/A	Patient education	Educate the patient about the do’s and don’ts of the condition. Make sure that the suture should not get wet. There should not be any oozing or pus formation around the suture site. Exercise regularly.	N/A	To promote faster healing of the suture site. To prevent any infection. To improve circulation around the suture site.
Pain and swelling	To decrease pain and swelling around the operated site	Cryotherapy (around the suture site) Precaution should be taken so that the suture should not get wet. Elevation and fist making, and opening [11].	20 minutes every four hourly	Desensitization leads to pain relief
Reduced ROM	To improve range of motion	For the wrist joint, use flexibility, extension, ulnar deviation, and radial deviation ROM exercises. For radioulnar joint - pronation, supination ROM exercises. For MCP joint - flexion, extension ROM exercises. For PIP and DIP joint - flexion, extension. Muscle energy technique for wrist joint (reciprocal inhibition) flexion, extension. Maitland grade 2 mobilizations.	8 repetitions × 1 set 30 oscillations × 3 sets	To help the patient do regular functional activity, improve the mobility of the joints, and improve the range of motion.
Reduced flexibility of wrist joint	To improve the flexibility of the left wrist joint	Stretching exercises [12]	30 seconds × 3 sets	By improving flexibility, the patient can perform activities of daily living effectively.
Reduced strength of the wrist joint	To increase strength	Isometric exercises for left wrist, forearm, MCP, DIP, and PIP joint	12 repetitions × 2 sets with (10 secs hold)	To improve the strength of the muscles
Scar	To make the scar adherent and pliable	Scar tissue mobilization longitudinal vertical [13]	15 minutes twice a day	It will help improve flexibility of the skin and fascia
Reduced grip strength	To improve grip strength of left hand	Lumbricals strengthening (fisting, towel crumbling exercise, and progress to Paper crumbling)	12 repetitions × 2 sets with (10 secs hold)	It will help the patient to perform daily ADLs with minimum difficulty.
N/A	Assistive device	Cock up splint [14]	Wean out every two hours for 15 mins. It should not be worn during the nighttime.	To prevent contracture and assist in movement.

**Figure 1:**
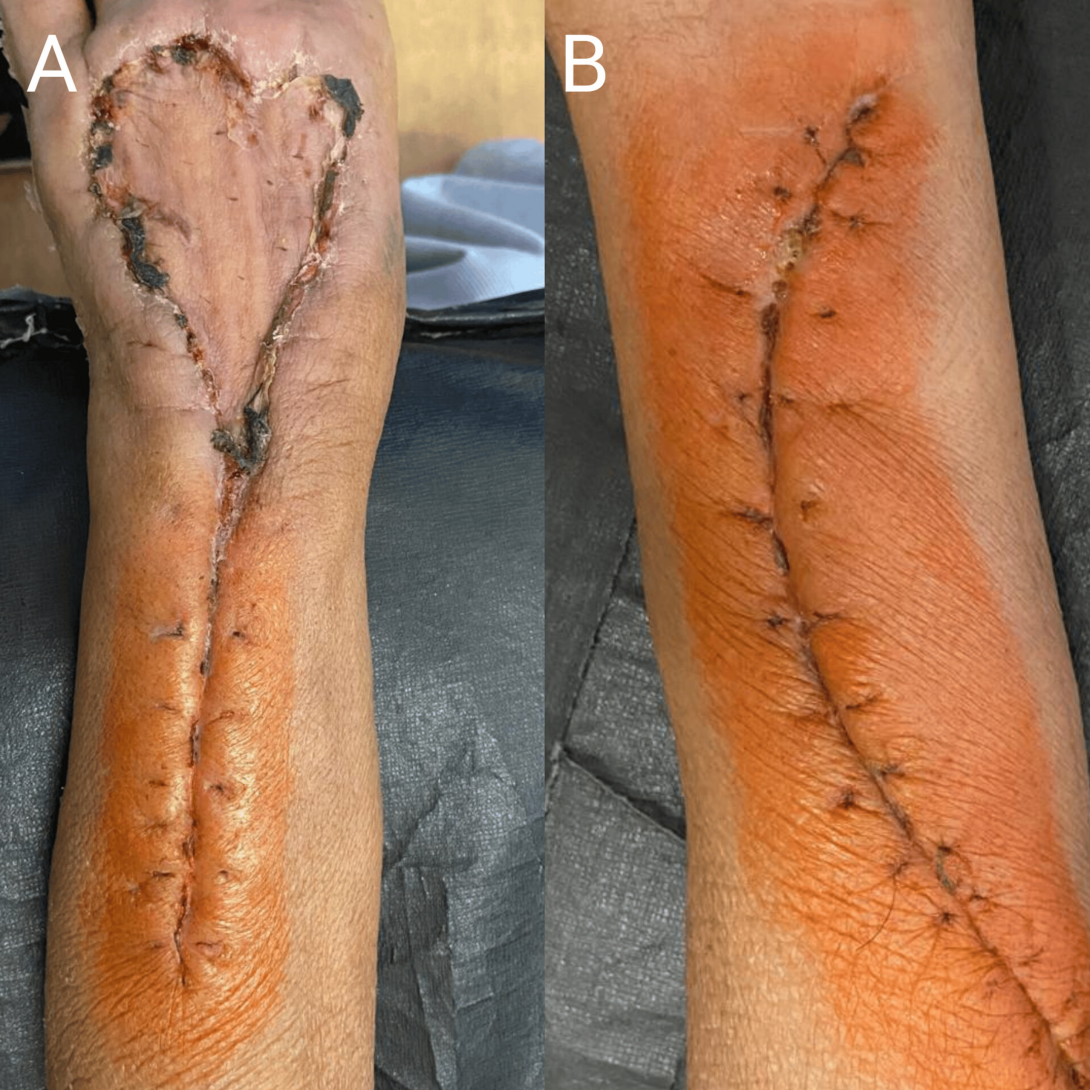
Pre-rehabilitation suture site. (A) The pre-rehabilitation suture site of the extensor aspect of the left forearm and the hand; (B) the pre-rehabilitation suture site of the flexor aspect of the left forearm and hand.

Patient performing exercise while therapist assisting the movement is demonstrated in Figure 1.

**Figure 2:**
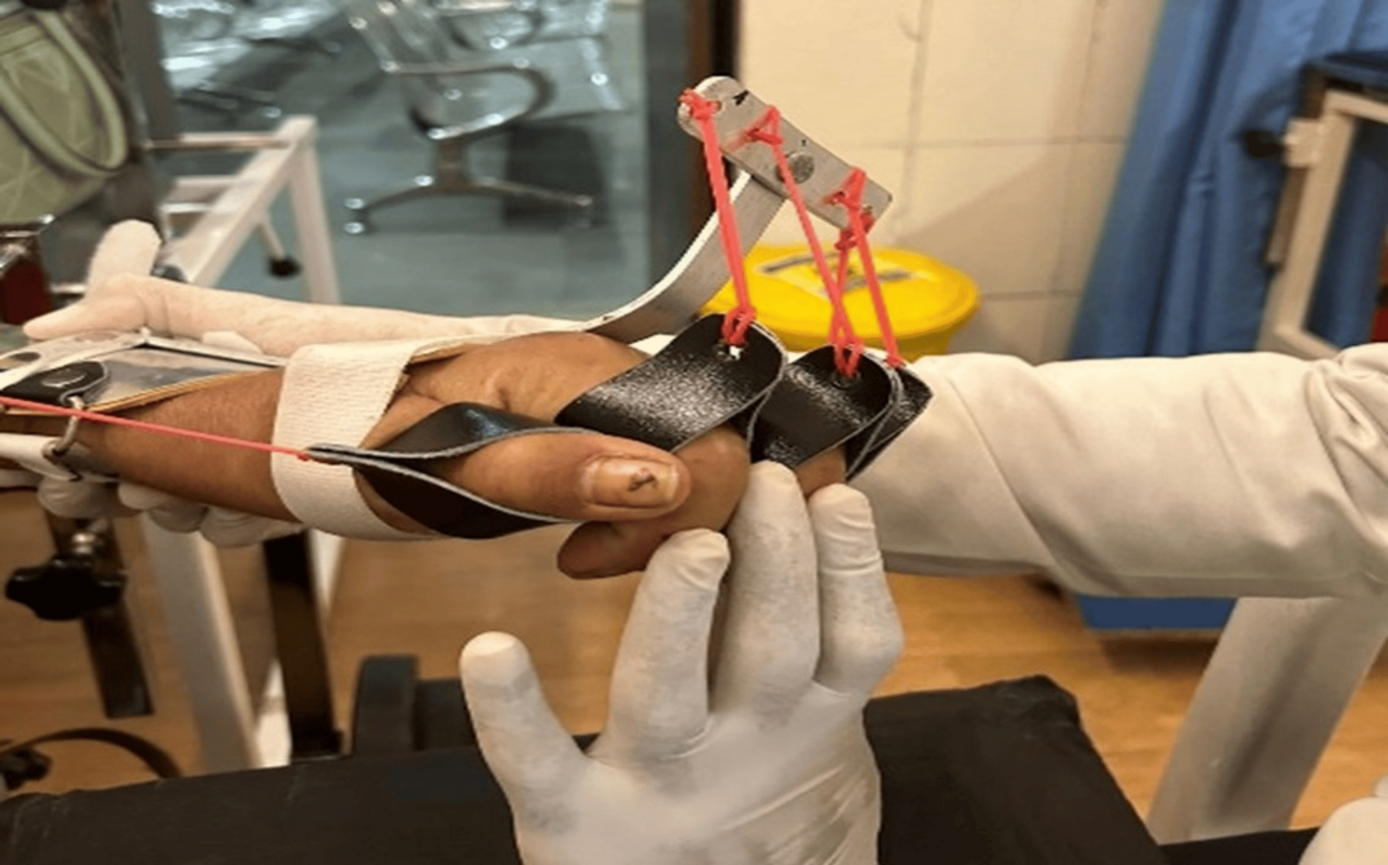
Cock-up splint.

**Figure 3:**
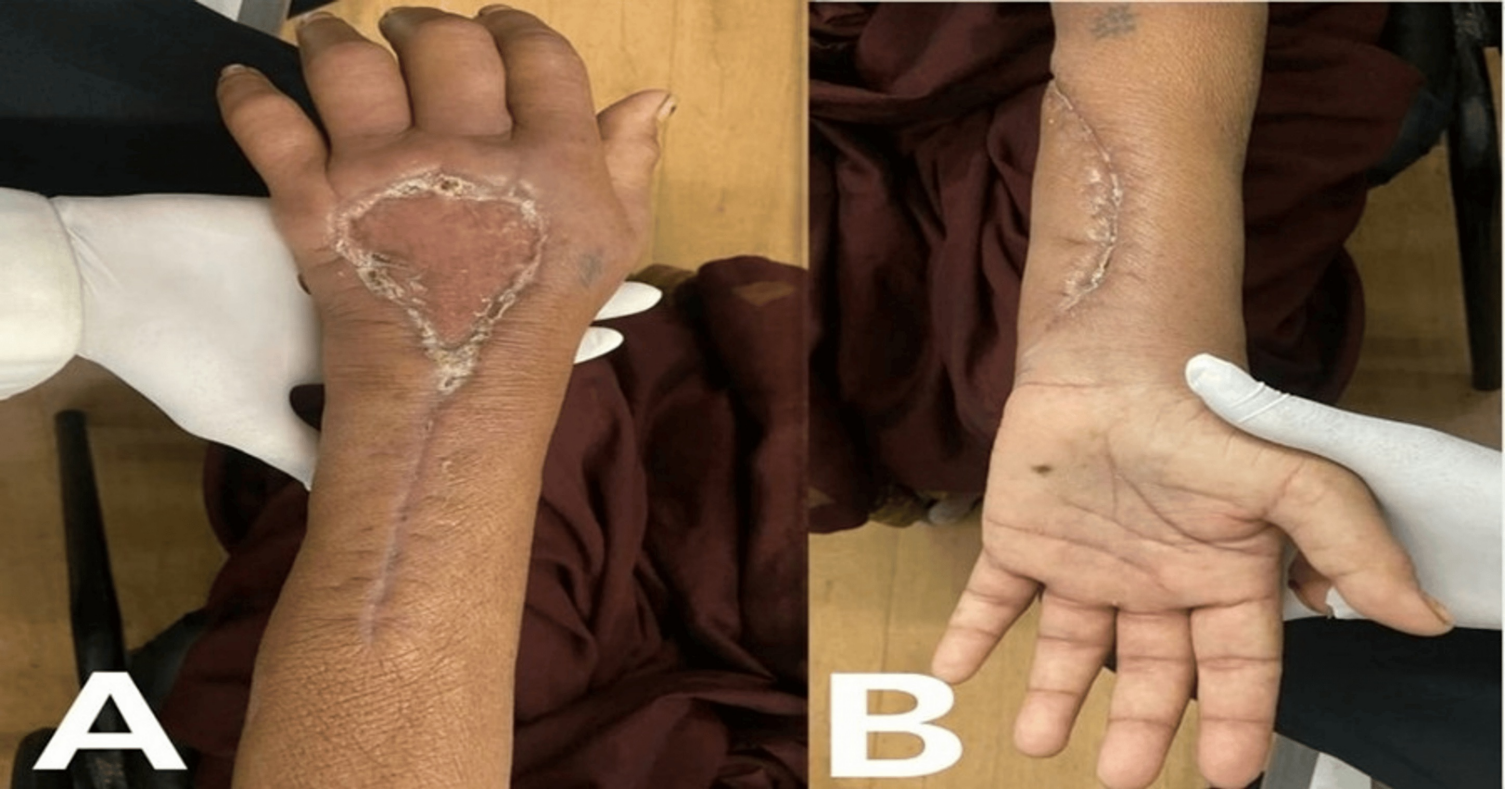
Post-rehabilitation suture site showing improvements in swelling, wound healing, and overall tissue condition. (A) Post-rehabilitation suture site of extensor aspect of left forearm and hand; (B) post-rehabilitation suture site flexor aspect of left forearm and hand.

The patient's hand was kept in a cockup splint, as shown in Figure 2. Figure 3 demonstrates the rehabilitation suture site of the ventral and dorsal surfaces of the forearm.

Range of motion and strength were examined after the physiotherapy treatment, whose results are depicted in Tables 4-5, respectively.

**Table 4:** Post-rehabilitation range of motion. MCP: metacarpophalangeal.

Range of Motion	Right	Left
Wrist flexion	0–60^◦^	0–55^◦^
Wrist extension	0–60^◦^	0–55^◦^
Ulnar deviation	0–30^◦^	0–25^◦^
Radial deviation	0–20^◦^	0–20^◦^
1^st ^MCP flexion	0–50^◦^	0–45^◦^
2^nd^ MCP flexion	0–80^◦^	0–70^◦^
3^rd ^MCP flexion	0–75^◦^	0–70^◦^
4^th^ MCP flexion	0–80^◦^	0–70^◦^
5^th^ MCP flexion	0–85^◦^	0–75^◦^
1^st ^MCP extension	50–0^◦^	45–0^◦^
2^nd^ MCP extension	80–0^◦^	70–0^◦^
3^rd^ MCP extension	75–0^◦^	65–0^◦^
4^th^ MCP extension	80–0^◦^	70–0^◦^
5^th^ MCP extension	85–0^◦^	75–0^◦^
Forearm supination	0–80^◦^	0–80^◦^
Forearm pronation	0–80^◦^	0–80^◦^

**Table 5:** Post-rehabilitation manual muscle testing. MCP: metacarpophalangeal.

Muscles	Right	Left
Wrist flexors	5\5	4\5
Wrist extensors	5\5	4\5
Ulnar deviators	5\5	4\5
Radial deviators	5\5	4\5
1^st^ MCP	5\5	4\5
2^nd^ MCP	5\5	4\5
3^rd ^MCP	5\5	4\5
4^th^ MCP	5\5	4\5
5^th^ MCP	5\5	4\5
Forearm pronators	5\5	4\5
Forearm supinators	5\5	4\5

Outcome measures

Pre- and post-treatment to assess the ability of the patient to perform his daily activities before and after physiotherapy rehabilitation, as illustrated in Table 6.

**Table 6:** Outcome measures. NPRS: numerical pain rating scale; DASH: disability of arm, shoulder and hand questionnaire is used as an indicator of the impact of an impairment on disability, the level in which 0% denotes “no disability” and 100% denotes “severe disability.”

Outcome	Pre-intervention	Post-intervention
NPRS	On movement: 09/10	On movement: 02/10
	At rest: 07/10	At rest: 01 /10
DASH	75.8%	27%

## Discussion

This case report emphasizes the role of physiotherapy in the rehabilitation process of a patient with fasciotomy for acute spreading cellulitis. Cellulitis is an acute bacterial infection that may be serious in nature and lead to compartment syndrome, hence requiring urgent surgical intervention in the form of a fasciotomy [15]. The rehabilitation of this patient underlines the need for a systematic regimen of physiotherapy to regain functional activity, range of motion, and muscle strength. Cellulitis presents with redness, swelling, and pain. It can further be divided into two types: purulent and non-purulent. The former is the one that presents with pus, and the pus needs to be drained along with antimicrobials as the mainstay of treatment. The patient developed acute compartment syndrome due to infection, for which urgent fasciotomy and split skin grafting were done [16]. Cryotherapy and elevation were applied for common postoperative pain and swelling. Elevation reduces oedema by improving venous return. Cryotherapy, on the other hand, does not only reduce inflammation but also provides analgesia to the affected area by numbing it. Range of motion exercises to the wrist, forearm, and MCP joints were performed to prevent stiffness and improve flexibility; both are pertinent in maintaining the functionality of the joints [17].

A case report has noted that physiotherapy can help improve the range of motion, promote joint health, and can also help in reducing joint stiffness in post-operative cases of fasciotomy presenting as intramuscular haematoma with compartment syndrome in haemophilia patients. All these improvements lead to increased functional independence. Scar tissue formation is a common complication after fasciotomies. Modalities such as massage and mobilization of the scar can be performed for up to four weeks in order to avoid excessive adhesion, maximize tissue flexibility, and relieve the pain of scar tissue formation. These strategies promote maximal recovery with the least amount of pain and suffering.

A study by Singh et al. showed that physiotherapeutic interventions improve scar development [18]. Aggressive physiotherapy, along with medical and surgical treatment instituted from the beginning, provides maximum improvement in functional outcomes [19].

## Conclusions

This case study emphasizes the need for physiotherapy in the post-fasciotomy recovery of patients with cellulitis-induced compartment syndrome. A well-structured physiotherapy program can help patients heal faster, regain functional capacity, and improve their overall quality of life. Early intervention, together with patient education and attention to prescribed exercises, is critical for best results. Additional research and case studies are required to enhance these methods and validate their efficacy across a wide range of patient demographics. The limited availability of published literature specific to this region meant that the physiotherapy approach utilized in this study was based on basic therapeutic principles.

## References

[1] Bystritsky RJ (2021). Cellulitis. Infect Dis Clin North Am.

[2] Robinson CA, Kellar JZ, Stehr RC (2021). An 84-Year-old man with acute atraumatic compartment syndrome of the upper extremity due to streptococcus pyogenes cellulitis. Am J Case Rep.

[3] Vignes S, Poizeau F, Dupuy A (2022). Cellulitis risk factors for patients with primary or secondary lymphedema. J Vasc Surg Venous Lymphat Disord.

[4] Dupuy A, Benchikhi H, Roujeau JC (1999). Risk factors for erysipelas of the leg (cellulitis): case-control study. BMJ.

[5] Hayeri MR, Ziai P, Shehata ML, Teytelboym OM, Huang BK (2016). Soft tissue infections and their imaging mimics: from cellulitis to necrotizing fasciitis. Radiographics.

[6] Dinia M, Bouzid B, Berrada MS (2023). Acute compartment syndrome of the upper extremity due to staphylococcus aureus cellulitis: an unusual etiology and dramatic consequence. Australas Medical J.

[7] Whitesides TE, Heckman MM (1996). Acute compartment syndrome: update on diagnosis and treatment. J Am Acad Orthop Surg.

[8] Rawat R, Lanjewar SM, Chauhan RK, Baghel J (2022). Factors affecting graft uptake of large wound surface, covered by mesh split skin grafting: a longitudinal study. J Clin Diagn Res.

[9] Hatwar V, Phansopkar P (2023). Physiotherapy rehabilitation following lower extremity split skin grafting in necrotizing fasciitis. Cureus.

[10] Kumar S, Sambyal S, Nagar A (2023). A case study on the effect of physiotherapy on joint health and functional independence in a person with hemophilia who underwent upper limb fasciotomy. Int J Med Res Health Sci.

[11] Schubert AG (2011). Exertional compartment syndrome: review of the literature and proposed rehabilitation guidelines following surgical release. Int J Sports Phys Ther.

[12] Flautt W, Miller J (2013). Post-surgical rehabilitation following fasciotomies for bilateral chronic exertional compartment syndrome in a special forces soldier: a case report. Int J Sports Phys Ther.

[13] Mauffrey C, Hak DJ, Martin MP (2019). Compartment Syndrome: A Guide to Diagnosis and Management [Internet]. https://pubmed.ncbi.nlm.nih.gov/32091680/.

[14] Howell DM, Bechmann S, Underwood PJ (2023). Wrist Splint. https://www.ncbi.nlm.nih.gov/books/NBK557630/.

[15] Wallace HA, Perera TB (2023). Necrotizing Fasciitis. https://www.ncbi.nlm.nih.gov/books/NBK430756/.

[16] Sullivan T, de Barra E (2018). Diagnosis and management of cellulitis. Clin Med (Lond).

[17] Cina-Tschumi B (2007). [Evidence-based impact of cryotherapy on postoperative pain, swelling, drainage and tolerance after orthopedic surgery]. Pflege.

[18] Singh R, Dinakaran M, Vandhiyadevan GD, Mathangi S, Pandey RA, John MJ (2021). Responsiveness to hemophilia joint health score and functional independence score in patients with hemophilia with intermittent factor support and physiotherapy. Chrismed J Health Res.

[19] Thomas DK, Budhoo EJ, Mencia MM, Ali TF, Santana D (2014). A case of upper limb compartment syndrome following snake envenomation measure twice, cut once. West Indian Med J.

